# Update on Neurological Manifestations of SARS-CoV-2

**DOI:** 10.5811/westjem.2020.8.48839

**Published:** 2020-10-06

**Authors:** Hisham M. Valiuddin, Almir Kalajdzic, James Rosati, Kevin Boehm, Dominique Hill

**Affiliations:** * University of Pennsylvania, Department of Emergency Medicine, Philadelphia, Pennsylvania; † St. Mary Mercy Hospital, Department of Emergency Medicine, Livonia, Michigan; ‡ Broward Health Medical Center, Department of Emergency Medicine, Fort Lauderdale, Florida

## Abstract

Severe acute respiratory syndrome coronavirus 2, the source of COVID-19, causes numerous clinical findings including respiratory and gastrointestinal findings. Evidence is now growing for increasing neurological symptoms. This is thought to be from direct in-situ effects in the olfactory bulb caused by the virus. Angiotensin-converting enzyme 2 receptors likely serve as a key receptor for cell entry for most coronaviridae as they are present in multiple organ tissues in the body, notably neurons, and in type 2 alveolar cells in the lung. Hematogenous spread to the nervous system has been described, with viral transmission along neuronal synapses in a retrograde fashion. The penetration of the virus to the central nervous system (CNS) allows for the resulting intracranial cytokine storm, which can result in a myriad of CNS complications. There have been reported cases of associated cerebrovascular accidents with large vessel occlusions, cerebral venous sinus thrombosis, posterior reversible encephalopathy syndrome, meningoencephalitis, acute necrotizing encephalopathy, epilepsy, and myasthenia gravis. Peripheral nervous system effects such as hyposmia, hypogeusia, ophthalmoparesis, Guillain-Barré syndrome, and motor peripheral neuropathy have also been reported. In this review, we update the clinical manifestations of COVID-19 concentrating on the neurological associations that have been described, including broad ranges in both central and peripheral nervous systems.

## INTRODUCTION

Severe acute respiratory syndrome coronavirus 2 (SARS-CoV-2), the source of coronavirus disease 2019 (COVID-19), causes numerous clinical findings including well described respiratory and gastrointestinal findings. While literature on SARS-CoV-2 association with neurological findings was initially sparse, evidence is now rapidly growing for this potentially devastating link. Vigilance is important to recognize all possible sequelae of COVID-19; additionally, early detection and recognition is a mainstay of medicine across any disease.

During the initial outbreak in Wuhan, China, a wide range of clinical presentations was found beyond the typical respiratory symptoms, with close to 50% of patients having gastrointestinal (GI) symptoms, and 7% of patients having no respiratory symptoms.^1^ While the US Centers for Disease Control and Prevention (CDC) definition of persons under investigation for COVID-19 has evolved, it generally includes the presence of fever and signs and symptoms of respiratory illness. While this may encompass a large number of cases, it also leaves a big gap in untested patients with minimal to no respiratory symptoms including those with only GI or neurologic symptoms. Earliest reports from Wuhan found that over 36% of patients had some degree of nervous system involvement, the most common being dysfunction of the central nervous system (CNS) with close to 15% of patients having complaints of dizziness or headache.^2^ In this article we provide a review of central and peripheral nervous system (PNS) involvement of SARS-CoV-2 (Table 1).

## METHODS

We conducted a literature review to obtain data regarding neurologic manifestations of COVID-19. All searches were done in May 2020 using Google searches, Google Scholar, and PubMed using combinations of the following keywords: “COVID,” “CNS,” “PNS,” “neurologic,” “coronavirus,” “manifestation,” “symptoms,” and “nervous.” Articles were initially selected based on their titles and abstracts for relevance to our review. Table 1 shows the case reports, reviews, and studies that were included in our review. We included articles that described central or peripheral nervous system neurological sequelae in patients with COVID-19. Articles were published between November 2019–May 2020. Exclusion criteria consisted of any articles that did not describe neurologic involvement of COVID-19. In total we included 26 articles in our review, which consisted of case reports and case series, as well as retrospective and prospective observational studies. We excluded 307 articles as not being pertinent to the neurological scope of this study. Also included in our review are articles that discuss potential mechanisms of neurologic involvement of COVID-19 to provide a better understanding of the disease process being described.

## DISCUSSION

SARS-CoV-1 from the early 2000s was found to have neurologic spread, with evidence of the virus isolated from cerebral spinal fluid (CSF) fluid.^3^ The route of entry to the brain appeared to be predominantly through olfactory bulb neurons.^3^ The intense systemic inflammatory response associated with viral infection can lead to blood-brain barrier breakdown, allowing cytokines access to the CNS.^4^,^5^,^6^ The penetration of the virus to the CNS allows for the resulting intracranial cytokine storm, which can result in complications such as acute necrotizing encephalopathy (ANE), CNS disturbances, headache, trouble walking, visual disturbances, weakness, and even stroke.^7^,^8^ This cytokine response can cause a myriad of hematologic issues ranging from thrombosis to a hemophagocytic lymphohistiocytosis.^9^

Transmission of the SARS-CoV-2 virus can occur via droplet and contact transmission as well as through airborne route under specific circumstances.^10^,^11^ SARS-CoV-2 can enter the CNS through the cribriform plate and cause neurologic symptoms.^5^,^6^ Initially, a group from France looked at the utility of using hyposmia and hypogeusia as a screening tool for COVID testing, and found that close to 20% of patients who tested positive for SARS-CoV02 had self-reported hyposmia and hypogeusia.^12^ This is thought to be from direct in-situ effects in the olfactory bulb caused by the virus. Due to the reported frequency of hyposmia and hypogeusia, the American Academy of Otolaryngology Head and Neck Surgery and the British Association of Otorhinolaryngology now recommend that these symptoms be added to the list of primary screening symptoms for COVID-19. However, this is not a finding that is unique to SARS-CoV-2, as many respiratory viruses have been associated with hyposmia and hypogeusia in the past.

**Population Health Research Capsule**

What do we already know about this issue?

*COVID-19 can severely affect many organ systems, including respiratory, gastrointestinal, and nervous*.

What was the research question?

What are the current known neurological manifestations of the SARS-CoV-2 virus?

What was the major finding of the study?

*Broad and diverse nervous system involvement, including central and peripheral nervous systems, have been identified*.

How does this improve population health?

*This review provides an increased awareness of the signs, symptoms, and presentations of neurological complications associated with COVID-19*.

In addition to the novel SARS-CoV2, other coronaviridae, enteroviridae, rhinoviridae, parainfluenza virus, and Epstein-Barr virus have all been associated with post viral olfactory dysfunction (PVOD).^13^ While the percentage of patients with a viral illness who develop olfactory dysfunction is unclear, the phenomenon is well described in the literature. Quint and colleagues looked at a series of 120 patients who had nonconductive olfactory disorders and found upper respiratory infection (URI) to be the most common cause, occurring in 42.5% of the patients.^14^ The olfactory dysfunction occurs in the acute symptomatic phase of the virus and then often persists for a prolonged period of time thereafter.^14^ The initial dysfunction could be attributed to mucosal edema, but in many cases the olfactory dysfunction persists.

When explored further, varying pathologies were identified. Douek and colleagues identified extensive scarring on biopsy as well as replacement of the olfactory epithelium with respiratory epithelium in patients with PVOD.^15^ Additionally, Jafek and colleagues found decreased numbers of olfactory receptors in patients with PVOD.^16^ Yamagishi and colleagues also identified decreased numbers of olfactory receptors and nerve bundles in post-URI olfactory loss.^17^, ^18^, ^19^ Early reports from Wuhan found that approximately 5% of patients had impairment of taste and smell, while later reports from Vaira et al demonstrate a much higher incidence, upwards of 19% of the 324 patients evaluated.^20^ Additionally, the reports out of Wuhan revealed 13% of patients had headaches, and in severe disease they noted acute cerebrovascular accident (CVA) presented in close to 6% of patients in the intensive care unit (ICU).^1^ Central and peripheral nervous system involvement has been described through a variety of presentations.

### Peripheral Nervous System

#### Guillain-Barré Syndrome

PNS findings include hyposmia and hypogeusia as discussed above, and there have been cases reported of Guillain-Barré syndrome (GBS). One case report described a confirmed COVID-19 patient who developed progressive bilateral ascending paralysis two weeks after developing respiratory symptoms.^21^ This patient had electromyography and neuronal testing that was consistent with GBS and was treated with intravenous immunoglobulin (IVIG) 0.40 grams per kilogram per day; however, the patient refused lumbar puncture for CSF analysis. No outcome post treatment was reported.

A case series of five COVID-19 patients with new diagnosis of GBS in Northern Italy did report post-IVIG outcome measures.^22^ All five of the patients were found to have CSF testing that showed less than five white blood cells per cubic millimeter, and negative reverse transcription polymerase chain reaction assay for SARS-CoV-2.^22^ All of these patients underwent IVIG treatment, two of whom received a second course of IVIG and a third who received plasma exchange.^22^ The outcomes reported that of the two patients who received a second course of IVIG, one remained in the ICU on mechanical ventilation at four weeks post-IVIG, while the other had bulbar symptom improvement although minimal improvement in extremity weakness. The patient who received plasmapheresis remained tetraplegic and ventilator dependent four weeks post treatment. Of the two patients who received only one course of IVIG, one who presented initially with only mild facial and upper extremity weakness had improvement of symptom and discharge; the other patient who presented with moderate to severe upper and lower extremity weakness was still unable to stand and was transferred to a rehabilitation center.

#### Bell’s Palsy

Mehta et al described a case of a 36-year-old patient who presented with complaint of numbness, tingling, and weakness of the right side of his face.^23^ This patient had fevers, chills, and myalgias for three days prior to his neurologic complaints. The right side of his forehead had no movement, and he was unable to close his right eye.^23^ Computed tomography (CT) angiogram of the head showed no abnormalities. He was diagnosed with Bell’s palsy, prescribed prednisone and eye lubrication, and discharged to an isolation shelter as the patient was homeless.^23^ His COVID-19 swab came back positive, and the patient was transferred further to a COVID-19 isolation shelter.^23^

Goh et al described a case of a patient with facial nerve palsy that developed in a 27-year-old patient on day 6 of his illness with COVID-19, while having been hospitalized for three days.^24^ The patient developed left-sided facial weakness that was preceded by left retroauricular pain and dysgeusia.^24^ He was started on prednisone and valacyclovir, as well as lopinavir/ritonavir in an attempt to reduce SARS-CoV-2 viral replication.^24^ After one week, the patient had no significant change in his facial nerve palsy symptoms.^24^

### Central Nervous System

#### Cerebrovascular Accidents

From a CNS standpoint, there appears to have been an increase in cases reported of CVA with large vessel occlusion in people younger than 50, with many of these patients testing positive for SARS-CoV2.^25^ Oxley et al found five cases of CVA in patients younger than 50 over a two-week period from March 23–April 7, 2020, with an average National Institutes of Health Stroke Score (NIHSS) of 17, indicating severe infarction. Researchers extracted data from every two-week period over the precedng 12 months and found the baseline rate of CVA in the mentioned age group was 0.73 patients in 14 days.^25^ In another study, Li et al performed a single-center, retrospective observational study, which revealed that of the 219 patients with SARS-CoV-2, 10 (4.6%) developed ischemic stroke. Of the patients who tested positive for SARS-CoV-2, they were more likely to have an increased inflammatory response as reflected by the elevated D-dimer (6.9 [0.3–20] vs 0.5 [0.1–20] milligrams per liter [mg/L], p<0.001), and C-reactive protein (51.1 [1.3–127.9] vs 12.1 [0.1–212] mg/L, p<0.05) in these patients compared to patients who did not have SARS-CoV-2.^26^

The Mao et al study revealed a similar finding but took this point further. In their retrospective observational case series, they defined the degree of severity of SARS-CoV-2 infection as severe vs non-severe using the American Thoracic Society guidelines for community-acquired pneumonia.^27^ Of the 214 patients who tested positive for SARS-Co-2, 88 patients had a severe infection and 126 had non-severe infection. Of those 88 patients, five (5.7%) developed cerebrovascular disease vs only one (0.8%) in those with non-severe infection.^2^ This suggests that infection with the virus in isolation is not the sole factor for developing cerebrovascular disease. Rather, the illness severity could be playing a role, and likely corresponds to an increased inflammatory state.^2^

#### Cerebral Venous Sinus Thrombosis

Hughes et al reported of cerebral venous sinus thrombosis, where the patient had presented with headache that progressed to right-sided weakness, numbness, and expressive aphasia with a NIHSS of 10, which was confirmed on CT venogram to be a sigmoid and transverse sinus thrombosis.^28^ This patient improved with low-molecular-weight heparin treatment and outpatient apixaban.

#### Acute Myelitis

There have been cases of acute myelitis, first reported in Wuhan in a patient who was admitted to medical ward for COVID-19, and subsequently developed acute bilateral lower extremity weakness, loss of sensation, hyporeflexia, and urinary incontinence.^29^ This patient was positive for SARS-Cov-2, and serologic testing for a plethora of other potential causative agents was negative. Of note, they did find that this patient also had developed CNS involvement, with basal ganglia and periventricular lacunar infarcts.^29^

#### Acute Necrotizing Encephalitis

There have been reported cases of ANE associated with SARS-CoV-2, which is caused by breakdown of the blood-brain barrier rather than direct viral invasion, providing another route of CNS sequelae, even when the virus does not invade the neuron.^7^,^9^ Radiographic manifestations for ANE include hemorrhagic rim-enhancing lesions within the bilateral thalami, medial temporal lobes, and subinsular regions on magnetic resonance imaging.^7^ These patients present with fever, cough, and profound altered mental status. ANE is not the only cause of altered mental status by COVID-19 as there are a growing number of reports and concerns about severe ICU delirium associated with the disease.

#### Delirium

Beyond the typical causes of ICU delirium, patients with COVID-19 are at even higher risk due to the extreme isolation from human contact.^30^ Early reports from Wuhan reported 7.5% of patients with delirium-like findings, but these were likely under-reported since 75% of cases are missed unless the patient is specifically evaluated for delirium.^2^,^30^

#### Parkinson’s Disease

Patients with underlying neurologic dysfunction such as those with Parkinson’s disease (PD) tend to have associated cardiovascular disease and respiratory dysfunction, which puts them at increased risk for developing severe COVID-19. Other comorbidities such as diabetes mellitus and CVA are often found in PD patients, which also places them at higher risk for developing severe COVID-19, given that PD patients on levodopa already have an independently higher risk of CVA.^31^ Dyspnea is found in 39% of PD patients, which is secondary to respiratory dysfunction because of respiratory muscle weakness, poor posture, and inadequate respiration excursions.^32^ Furthermore, these patients have impaired mastication and swallowing reflexes, leaving them more likely to develop aspiration pneumonia. The combination of these factors along with the neurodegeneration of the medulla’s respiratory center, which also can be attacked by SARS-CoV-2, places the PD patient at higher risk for developing more severe pneumonia and ultimately respiratory failure.^33^ Also, Parkinsonian hyperpyrexia syndrome, a movement disorder emergency, has been seen in PD patients with COVID-19 due to the combination of fever and altered dopaminergic medication intake.^34^ Although the patients experiencing this phenomenon may recover from COVID-19, some are left with significant disability, while others may not survive.

### Other Neurologic Sequelae

The aforementioned pathologies, and those listed in the accompanying table, demonstrate the broad range of neurological sequelae that have been described in the literature. Pathologies that have morbid outcomes, within the setting of potential treatment, were further expanded above. As more is revealed about COVID-19, the table will likely need further expansion of associated complications.

### Mechanism

There are numerous theories on the potential causative mechanisms of the neurological sequelae, including the discussed olfactory bulb transmission pathway. Angiotensin-converting enzyme 2 (ACE-2) is a key receptor for cell entry for most coronaviridae, including SARS-CoV-2, and it is present in multiple organ tissues in the body, notably neurons, smooth muscle cells and hepatocytes, with significantly high concentrations in type 2 alveolar cells in the lung.^35^ This explains why the virus predominates with respiratory symptoms, especially in the earlier stages. Hematogenous spread to the nervous system has been described, with viral transmission along neuronal synapses in a retrograde fashion.^35^ This has been found in other coronaviridae, with viral transmission through the neuron via exocytosis and subsequent binding on ACE-2 receptors, propagating along neuronal channels into the CNS.^35^ Neuro-invasion by SARS-CoV-2 is postulated to be at least partially responsible for exacerbating the acute respiratory failure patients with COVID-19 development.^33^

## LIMITATIONS

This review has several limitations. Most important is that correlation does not equal causation. As the patients infected with SARS-CoV-2 developed a neurologic complication, the pathophysiology of the virus is unknown. Additionally, the findings described may be attributed to systemic critical illness rather than the etiologic virus specifically. Another limitation is that patients with neurologic symptoms in isolation of cough or fever were not widely tested for SARS-CoV-2 as per CDC guidelines, which have been rapidly evolving. This could in fact lower detection for further cases of neurologic manifestations in the context of a SARS-CoV-2 infection. Lastly, none of the studies reviewed in this article had a control group. There is no evidence in literature yet to identify whether a greater incidence of neurologic manifestations exist with SARS-CoV-2 compared to the inherent risks of developing these neurologic diseases in a native population.

## CONCLUSION

While the respiratory manifestations caused by the SARS-CoV-2 virus, including significant progression to acute respiratory distress syndrome, are well described, there is a growing body of evidence describing multiorgan involvement, including neurologic sequelae from the virus. While anosmia and dysgeusia have been well documented as diagnostic symptoms from SARS-CoV-2, other peripheral system manifestations such as Guillain-Barré syndrome and ophthalmoparesis have also been seen. A wide spectrum of central nervous system manifestations has been observed from acute necrotizing encephalitis to transverse myelitis. In this review, we update the clinical manifestations of COVID-19 concentrating on the neurological associations that have been described so far, including broad ranges in both central and peripheral nervous systems.

## Figures and Tables

**Table 1:** List of the largest studies and respective study methodology of recently reported neurological pathologies associated with SARS-CoV-2.

Pathology	Level of evidence	Author
Central		

Large Vessel Occlusion - Cerebrovascular Accident	Retrospective observational study	Mao L. et al^2^
	Case series (5 patients)	Oxley T. et al^25^
	Retrospective observational study	Li Y. et al^26^
	Case series (10 patients)	Berekashvili et al^36^
	Prospective observational study	Lodigiani et al^37^
Transverse Myelitis	Case report	Zhao K. et al^29^
Seizure	Retrospective observational study	Somani et al^38^
	Case series (22 patients)	Galanopolou et al^39^
	Case report	Vollono et al^40^
Myasthenia Gravis	Case series (5 patients)	Anand et al^41^
Acute Necrotizing Encephalopathy	Case report	Poyiadji N. et al^7^
Acute Disseminated Encephalomyelitis	Case report	Zhang T. et al^42^
Encephalitis/ Meningoencephalitis	Case report	Moriguchi T. et al^43^
	Case report	Lorenz et al^44^
Corticospinal Tract Signs	Observational series (58 patients)	Helms J. et al^45^
Posterior Reversible Encephalopathy Syndrome	Case report	Kaya et al^46^
	Post mortem study	Coolen et al^47^
Cerebral Venous Sinus Thrombosis	Case report	Hughes et al^28^

Peripheral		

Anosmia and Dysgeusia	Retrospective observational study	Mao L. et al^2^
	Cross sectional study	Lee et al^48^
	Cross sectional study	Yan et al^49^
Motor Peripheral Neuropathy	Case report	Abdelnour et al^50^
Guillain-Barré	Case series (5 patients)	Toscano G. et al^22^
	Case report	Zhao H. et al^29^
	Case report	Virani et al^51^
Ophthalmoparesis	Case Series (2 patients)	Dinkin et al^52^
Bell’s Palsy	Case report	Mehta et al.^23^
	Case report	Goh et al.^24^
